# Determination of adapalene in gel formulation by conventional and derivative synchronous fluorimetric approaches. Application to stability studies and in vitro diffusion test

**DOI:** 10.1186/s13065-016-0181-0

**Published:** 2016-05-28

**Authors:** M. M. Tolba, R. M. El-Gamal

**Affiliations:** Department of Analytical Chemistry, Faculty of Pharmacy, University of Mansoura, Mansoura, 35516 Egypt

## Abstract

**Background:**

Adapalene is a retinoid analogue with actions similar to those of tretinoin. It is used in topical treatment of mild to moderate acne. A survey of the literature reveals that no spectrofluorimetric method has been reported yet for determination of ADP, so it was thought necessary to develop a highly sensitive stability indicating spectrofluorimetric method.

**Results:**

Two highly sensitive spectrofluorimetric approaches were conducted for the assay of adapalene (ADP) in its gel. In the first approach, ADP exhibits an intense native fluorescence at 389 nm after excitation at 312 nm using borate buffer (pH 7.0)/ethanol system. This approach was successfully applied for routine analysis of ADP in its gel and ideally suited to the in vitro diffusion test. To elucidate the inherent stability of ADP, bulk sample was subjected to different stress conditions as specified by ICH guidelines. The acidic and oxidative degradation products were resolved from the intact drug using second and first derivative synchronous fluorimetry at 346 and 312.45 nm, respectively (the second approach). The synchronous fluorescence was scanned at Δ λ of 80 nm in case of acidic degradation and at Δ λ of 100 nm in case of oxidative degradation. Good linearity was obtained for ADP over the range 2.0–14.0 ng/mL with good correlation coefficient   0.999 in each approach. The approaches were carefully examined in terms of linearity, accuracy and precision. They were suitable for routine quality control laboratory. Moreover, the stability-indicating power of the second approach was ascertained via forced degradation studies.

**Conclusions:**

The proposed approaches were validated and successfully applied for the quantitative assay of a small concentration of ADP in its pharmaceutical gel. The conventional spectrofluorimetry was ideally suited for in vitro diffusion test. Stability studies were also conducted using different forced degradation condition according to ICH recommendation.

**Graphical abstract:**
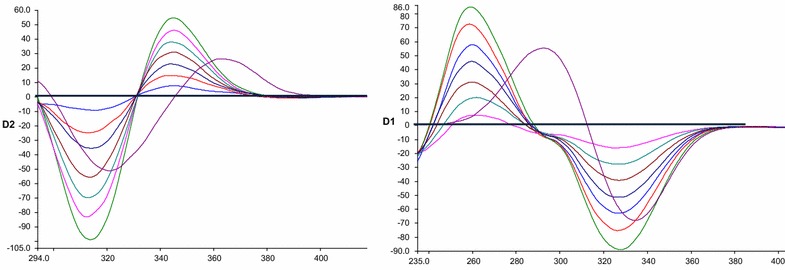
Simultaneous determination of ADP and its degradation products.

## Background

**Fig. 1:**
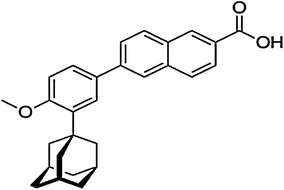
The structural formula for adapalene (ADP)

Chemically, adapalene (ADP) is 6-[3-(1-Adamantyl)-4-methoxyphenyl]-2-naphthoic acid (Fig. 1). It is a naphthoic acid derivative and retinoid analogue with actions similar to those of tretinoin. It is used in topical treatment of mild to moderate acne [1]. ADP is a subject of monograph in European Pharmacopoeia [2].

Only few analytical methods were reported for the assay of ADP. These methods include high performance liquid chromatography (HPLC) [3–8]. In addition, only two derivative spectrophotometric methods were applied for ADP determination in bulk drug and pharmaceutical dosage form [9] or in liposomes [10].

International Conference on Harmonization (ICH) guideline Q1A on stability testing of new drug substances and products requires that stress testing be carried out to elucidate the inherent stability features of the active substance which may be changed during storage and so, ensure high quality, safety, and efficacy of the pharmaceutical product [11].

Moreover, the development of in vitro release study serves as a good quality control tool to ensure batch to batch uniformity and screen experimental formulation during the product development. Determination of the value of in vitro release helps to cross check the product quality and product comparison [12].

A comprehensive literature survey revealed that no spectrofluorimetric method has been reported yet for the determination of ADP in its gel or in presence of its degradation products. The reported methods concerned with the stability of ADP are expensive, time consuming, sophisticated HPLC techniques [3–6]. Most of these methods suffer from low sensitivity which restricted the determination of ADP in low concentration in presence of its degradation products. Moreover, some of these methods showed narrow linearity range [5, 6] or failed to separate the acidic and oxidative degradation products from the parent drug [3, 6]. Regarding the pharmaceutical application, none of these methods are applicable to in vitro dissolution test which is an important issue in quality control laboratories.

Therefore, it was thought necessary to develop sensitive stability indicating spectrofluorimetric method for determination of ADP and applicable to in vitro diffusion test.

In our study, two extremely sensitive spectrofluorimetric approaches were explored for the analysis of a very small concentration of ADP down to 2.0 ng/mL. ADP shows a strong native fluorescence at 389/312 nm (λ_em_/λ_ex_) in borate buffer (pH 7.0)/ethanol system. Depending on this fact, the first approach was conducted and extended to study the inherent stability of ADP and the in vitro diffusion test. Great overlapping between the fluorescence spectra of ADP and its degradation products were observed, therefore, we resorted to derivative synchronous fluorimetry (DSF). Where, ADP was resolved from its acidic and oxidative degradation products by second (SDSF) and first (FDSF) derivative synchronous fluorimetry at 346 and 312.45 nm, respectively.

## Experimental

### Apparatus

- All fluorescence measurements were recorded with a Perkin-Elmer UK model LS 45 luminescence spectrometer, equipped with a 150 W Xenon arc lamp, grating excitation and emission monochromators and a Perkin Elmer recorder. The slit widths were 10 nm for both excitation and emission, and the photomultiplier voltage was set to automatic option. Derivative spectra were obtained using fluorescence data manager software, FL WINLAB, Version 4.00.02, Copyright 2001, Perkin Elmer, Inc., UK.
- A Consort P-901 pH-meter was used for pH measurements.
- Thermostatically controlled shaking water bath (Grant instrument Cambridge Ltd., Barrington Cambridge B2, 5002, England).
- Modified Franz diffusion cell.
- CAMAG UV-lamp, S/N 29000, dual wavelength 254/366 nm, 2 × 8 Watt (Switzerland) was used in the UV-degradation study.

### Materials and reagents

All the chemicals used were of analytical reagent grade, and the solvents were of HPLC grade.

- Adapalene was supplied by Glenmark (Cairo, Egypt) with a certified purity of 99.70 % and was used as received without further purification.
- Adapalene^®^ gel; batch # 011371, labeled to contain 0.1 % ADP (product of Borg Pharmaceutical Ind., Alexandria, Egypt). It was purchased from the local pharmacy.
- Ethanol (Fisher Scientific UK, Loughborough, Leics, UK).
- Acetonitrile, n-propanol and methanol were obtained from Tedia (USA).
- Boric acid, sodium acetate trihydrate, acetic acid 96 %, acetone, dimethyl formamide (DMF), methyl cellulose (MC), tween-80, sodium hydroxide, hydrogen peroxide (30 %) and hydrochloric acid (32 %) were all obtained from El-Nasr Pharmaceutical Chemicals Company (ADWIC) (Abu Zaabal, Egypt).
- Sodium dodecyl sulphate (SDS; 95 %), β-cyclodextrin (β-CD), cetrimide (CTAB; 99 %) were purchased from Winlab (UK).

### Standard solutions

Stock solution equivalent to 100.0 µg/mL of ADP was prepared by dissolving 10.0 mg in 100.0 mL ethanol. Other standard solution equivalent to 100.0 ng/mL was prepared by appropriate dilution of the stock solution with the same solvent. The solutions were found to be stable for at least 7 days without alteration when kept in the refrigerator.

### Procedures

#### Construction of calibration graphs

Aliquots of ADP standard solution were transferred into a series of 10 mL volumetric flasks so that the final concentration was in the range of 2.0–14.0 ng/mL. Then, 2 mL borate buffer (0.2 M, pH 7.0) was added to each flask followed by completing the volume with ethanol and mixing well. For the first approach, the fluorescence intensities of the solutions were measured at 389 nm after excitation at 312 nm. While, the second approach involved recording the synchronous fluorescence spectra of the solutions by scanning at Δ λ = 100 nm and Δ λ = 80 nm in case of acidic and oxidative degradation, respectively. The second and first derivative synchronous fluorescence spectra were derived. The peak amplitudes of the second (^2^D) or the first (^1^D) derivative spectra were estimated at 346 nm and 312.45 nm for acidic and oxidative degradation, respectively. A blank experiment was performed simultaneously. The relative fluorescence intensity (RFI) or the peak amplitude of the second (^2^D) or first (^1^D) derivative technique was then plotted against the final drug concentration in ng/mL to get the calibration graphs.

#### Analysis of ADP in semisolid pharmaceutical gel

An accurately weighed amount of the gel (0.1 g) was transferred into a clean dry 100 mL beaker and about 80 mL of ethanol was added. The flasks were placed in a water bath at 50 °C for 15 min followed by cooling. The contents were quantitatively transferred into 100 mL volumetric flask, completed to the mark with the same solvent and filtered through cellulose acetate syringe filter. The subsequent dilutions were performed via diluting an appropriate volume of this solution with ethanol and the procedures described under *“construction of calibration graphs”* were then applied. The nominal content was determined either from the previously plotted calibration graph or using the corresponding regression equation.

#### Procedure for in vitro diffusion test

The release of ADP in 65 % hydroethanolic solution was carried out using a modified diffusion cell according to the method adopted by Deo et al. [12].

The donor half-cell consists simply of a glass tube with an open end (3 cm in diameter) on which a semipermeable cellulose membrane was stretched and fixed by rubber band to prevent leakage of water.

Five grams of Adapalene^®^ gel were accurately weighed and thoroughly spread on the membrane to occupy 3 cm diameter circle. The donor cells were then immersed upside-down in 250 mL beaker containing 50 mL hydroethanolic solution (65 %) (receptor compartment) which was preheated and maintained at 37 ± 1 °C using thermostatically controlled water bath. The tubes height was adjusted, so that the membrane was just below the surface of the release medium. The whole assembly was shaken at 25 strokes per minute during the entire time of diffusion.

At the specified time interval, 2 mL was withdrawn from the receiver compartment and replaced by equal volume of fresh hydroethanolic solution and thus keeping a constant volume. Dilution of 0.1 mL was performed with ethanol up to 10 mL in a volumetric flask. Appropriate volumes (0.1 mL) of this solution were then transferred to 10 mL volumetric flask, 2 mL of 0.2 M borate buffer (pH 7.0) was added and the volume was completed to the mark with ethanol. The % released amounts of ADP were determined by conventional fluorimetry at 389 nm after excitation at 312 nm. Triplicate experiments were carried out for each sample.

#### Preparation of the degradation products

For degradation studies, working solution equivalent to 0.5 µg/mL was prepared by appropriate dilution of the stock solution with ethanol. Aliquots of 5 mL of this solution (equivalent to 2.5 µg) were then transferred into series of small conical flasks for alkaline, acidic and oxidative degradation. Then the following steps were performed:

##### For alkaline and acidic degradation

Aliquots of 5 mL of 2 M NaOH or different molarities of HCl (0.2–1 M) were added to the flasks. The solutions were heated in a boiling water bath under reflux for different time intervals (10–60 min). At the specified time, the contents of each tube were cooled, neutralized to pH 7.0 and the solutions were then transferred into a series of 25 mL volumetric flasks. The volumes were completed with ethanol. Aliquots of these solutions (1.4 mL) were transferred into a series of 10 mL volumetric flasks, followed by addition of 2.0 mL of 0.2 M borate buffer (pH 7.0) and completing the volumes to the mark using ethanol. The procedure under *“construction of calibration graphs”* for the first approach was then conducted.

##### For oxidative degradation

Five milliliters of 5–30 % H_2_O_2_ were added to each flask. The solutions were then heated in a thermostatically controlled water bath at 80 °C for different time intervals (10–60 min). At the specified time intervals, the contents of each flask were cooled and transferred to 25 mL volumetric flask. The volumes were completed to the mark with ethanol. The procedure mentioned under *“construction of calibration graphs”* for the first approach was then applied.

##### For day and UV light degradation

Suitable aliquots of the working solution (equivalent to 2.5 µg) were transferred into 25 mL volumetric flasks and completed to volume with ethanol. The flasks were left in day light or exposed to UV-light at 254 and 366 nm for 12 h in a wooden cabinet, where the distance between the source and the sample solution was kept at 15 cm. Aliquots of the solution (1.4 mL) were transferred into a series of 10 mL volumetric flasks and the procedure under ‘‘*construction of calibration graph*s’’ was then applied.

## Results and discussion

**Fig. 2:**
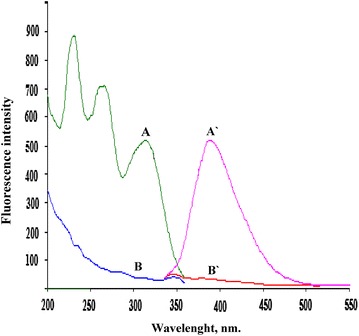
Fluorescence spectra of: *A*, *A*′ ADP (14.0 ng/mL) in borate buffer (pH 7.0)/ethanol system. *B*, *B*′ Blank (borate buffer (pH 7.0)/ethanol system) where: (*A*, *B*) Excitation spectra. (*A*′, *B*′) Emission spectra

From scanning the fluorescence spectra of ADP, it was found that ADP showed three different excitation peaks at wavelengths of 230, 265 and 312 nm and only one emission peak at 389 nm. In selection of excitation wavelength, we emphasize on the linearity and reproducibility of the calibration graph even if the other excitation wavelengths showed higher sensitivity relative to the selected one. Consequently, ADP fluorescence was measured at 389 nm after excitation at 312 nm (Fig. 2). Fortunately, the approach was extremely sensitive and allowed the determination of ADP in its pharmaceutical gel as alternative to the reported sophisticated HPLC methods. The high precision and accuracy of the approach made it ideal for in vitro diffusion test. Moreover, the inherent stability of ADP was investigated using the forced degradation studies.

**Fig. 3:**
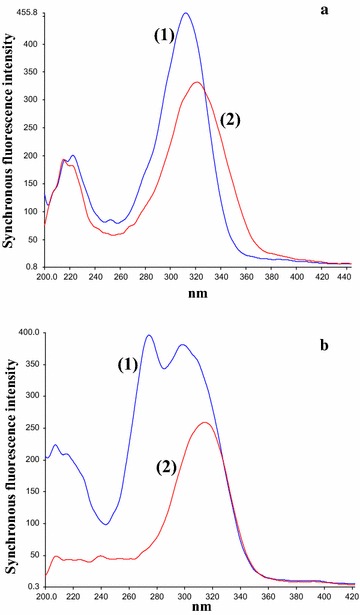
Synchronous fluorescence spectra of: (**a)** (*1*) ADP (14.0 ng/mL) (*2*) acidic degradation product; at Δ λ = 80 nm (**b**) (*1*) ADP (14.0 ng/mL) (*2*) oxidative degradation product; at Δλ = 100 nm

**Fig. 4:**
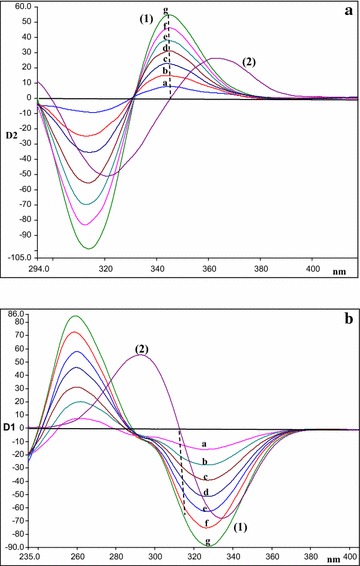
Second and first derivative synchronous fluorescence spectra of: (**a**) (*1*) (a–g) of ADP (2.0, 4.0, 6.0, 8.0, 10.0, 12.0, 14.0 ng/mL) at 346 nm (*2*) acidic degradation product (**b**) (*1*) (a–g) of ADP (2.0, 4.0, 6.0, 8.0, 10.0, 12.0, 14.0 ng/mL) at 312.45 nm (*2*) oxidative degradation product

After preliminary studies, neither conventional nor synchronous fluorimetry was able to resolve ADP from the degradation bands (Figs. 2, 3). Accordingly, we resorted to derivative synchronous fluorimetry which efficiently separated ADP from its acidic degradation product using SDSF and allowed its quantitation at 346 nm (Fig. 4a). Similarly, ADP was determined at 312.45 nm after application of FDSF to resolve its band from the oxidative degradation product (Fig. 4b). Thus, the stability-indicating power of this approach was ascertained.

### Optimization of experimental conditions

Various experimental parameters affecting the fluorescence intensities of ADP were investigated to achieve the maximum sensitivity and the ultimate selectivity.

#### Effect of pH

**Fig. 5:**
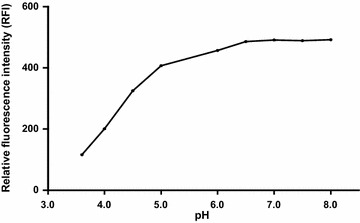
Effect of pH on the native fluorescence intensity of ADP (14.0 ng/mL)

To study the influence of pH on the fluorescence behavior of ADP, different types of buffers covering the whole pH range were tested, such as 0.2 M acetate buffer (pH 3.6–6) and 0.2 M borate buffer (pH 6.5–10), in addition to 0.1 M HCl and 0.1 M NaOH (Fig. 5). It was noticed that increasing the pH resulted in a proportional increase in the RFI of the drug up to 6.5, and then remained constant up to pH 8.0, after which a precipitation occurred at pH 9 and 10. Therefore, borate buffer pH 7.0 was selected as the optimum pH giving the highest sensitivity. Also, trials were made by replacement of buffer by 0.1 M HCl or NaOH. Indeed, utilizing 0.1 M NaOH resulted in a high RFI but almost equal to the fluorescence intensity achieved upon utilizing 0.2 M borate buffer with pH 7.0. And as a well-known fact that using buffer is more favorable to resist changes in pH values, so borate buffer was selected. On the contrary, using 0.1 M HCl showed a marked quenching of the RFI of ADP which may be attributed to its degradation.

#### Effect of diluting solvent

Various solvents including water, methanol, acetonitrile, ethanol, n-propanol, dimethyl formamide (DMF) and acetone were tested to choose the most convenient diluting solvent. It is noticeable from Table 1 that ethanol gave the highest fluorescence intensity so; it was the solvent of choice. Also, n-propanol could be used. High blank reading was observed in case of using methanol at this wavelength. Water and acetonitrile showed a marked decrease in the fluorescence intensity so they were not selected. The initiated intersystem crossing process caused by DMF made it unsuitable for ADP determination as it resulted in a marked decrease in the fluorescence intensity of ADP in addition to high blank reading [13]. Complete quenching of the fluorescence intensity was attained upon using acetone.

**Table 1:** Effect of diluting solvents on the relative fluorescence intensity of adapalene (14.0 ng/mL)

Diluting solvents	Relative fluorescence intensity (RFI)
Ethanol	492
n-propanol	451
Methanol	450
Water	124
Acetonitrile	165
Dimethyl formamide	51
Acetone	1

#### Effect of surfactant

**Fig. 6:**
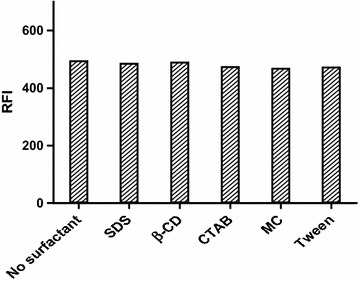
Effect of surfactant on the native fluorescence intensity of ADP (14.0 ng/mL)

Study of the impact of the surfactant was accomplished using 0.5 % aqueous solutions of anionic surfactant (SDS), cationic surfactant (CTAB), non-ionic surfactant (tween-80) and different macromolecules such as methyl cellulose, and β-CD. As shown in Fig. 6, these surfactants didn’t significantly affect the fluorescence intensity of ADP. Consequently, they were not incorporated in the procedure.

#### Selection of optimum Δ λ in the second approach

Selection of optimum Δ λ is a very essential criterion which should be considered during scanning of the synchronous fluorimetry as it may significantly affect sensitivity, resolution and symmetry of the bands. Consequently, a wide range of Δ λ (20–140 nm) was investigated. Good band shapes and adequate sensitivity were obtained upon using Δ λ of 80 and 100 nm in case of acidic and oxidative degradation, respectively. Lower and higher values of Δ λ than the optimum ones showed low fluorescence intensity for ADP and its degradation products. However, very low and very high Δ λ values caused irregularities in the spectral shape.

### Validation of the approaches

#### Linearity

It was investigated via replicate analysis of seven standard concentrations of ADP; 2.0, 4.0, 6.0, 8.0, 10.0, 12.0, 14.0 ng/mL. Calibration graphs of ADP were constructed by plotting either the RFI or the peak amplitude of (^2^D) or (^1^D) against the drug concentration in ng/mL. The results of the regression equations and correlation coefficients were abridged in Table 2. In the approaches, good linearity for ADP was achieved in the range of 2.0–14.0 ng/mL as indicated by higher value of correlation coefficients (>0.999).

**Table 2:** Analytical performance data for the proposed approaches

Parameters	The first approach at 389 nm	The second approach	
		SDSF at 346 nm	FDSF at 312.45 nm
Linearity range (ng/mL)	2.0–14.0	2.0–14.0	2.0–14.0
Intercept (*a*)	−4.714	−1.212	5.171
Slope (*b*)	35.357	3.975	3.729
Correlation coefficient (*r*)	0.9999	0.9998	0.9998
SD of residuals (S_*y/x*_)	1.59	0.34	0.31
SD of intercept (S_*a*_)	1.34	0.29	0.26
SD of slope (S_*b*_)	0.15	0.03	0.03
Percentage relative standard deviation, % RSD	0.42	0.88	0.76
Percentage relative error, *%* error	0.16	0.33	0.29
Limit of detection, LOD (ng/mL)	0.13	0.24	0.23
Limit of quantitation, LOQ (ng/mL)	0.38	0.73	0.69

#### Limit of quantitation (LOQ) and limit of detection (LOD)

These analytical parameters were computed by the equations specified by ICH Q2R1 recommendations [14] and were presented in Table 2:

$${\rm{LOQ}} = 10 \,{\rm{S}}_{\rm{a}}/{\rm{b}} \quad {\rm{and}} \quad{\rm{LOD}} = 3.3 \,{\rm{S}}_{\rm{a}}/{\rm{b}}$$

where S_a_ = standard deviation of the intercept of the calibration curve and b = slope of the calibration curve.

#### Accuracy and precision

The results of the present approaches were statistically compared with those of the comparison method [3] to ascertain these analytical features. No significant difference was observed using Student’s *t* test and variance ratio F test [15]. The excellent recovery values demonstrated that the approaches were sufficiently accurate over the specified range (Table 3).

**Table 3:** Application of the proposed approaches for the determination of adapalene in pure form

Parameter	Amount taken (ng/mL)	First approach at 389 nm		Second approach				Comparison method [3]	
		Amount found (ng/mL)	% found	Amount found (ng/mL)		% found		Amount found (µg/mL)	% found
				SDSF at 346 nm	FDSF at 312.45 nm	SDSF at 346 nm	FDSF at 312.45 nm		
ADP	2.0	2.00	100.00	1.991	1.992	99.54	99.62	20.0	99.22
	4.0	4.008	100.20	4.001	3.977	100.02	99.43	30.0	99.88
	6.0	5.988	99.80	5.976	5.962	99.59	99.36	40.0	101.15
	8.0	8.053	100.66	8.134	8.000	101.68	100.00		
	10.0	9.948	99.48	9.915	10.146	99.15	101.46		
	12.0	11.956	99.63	11.918	12.023	99.31	100.19		
	14.0	14.049	100.35	14.066	13.900	100.47	99.29		
Mean			100.02			99.97	99.91		100.08
SD			0.42			0.88	0.76		0.98
*t*			1.06 (2.31)*			0.73 (2.31)	0.93 (2.31)		
*F*			2.88 (5.14)*			1.53 (19.33)	1.16 (19.33)		

*N.B.* Each result is the average of three separate determinations

* The values between parentheses are the tabulated *t* and *F* values at *P* = 0.05 [15]

The comparison HPLC method [3] was carried out on C8 column using a blend of methanol: ammonium acetate buffer pH 4.0 (80:20, v/v) as a mobile phase and UV detection at 270 nm.

The repeatability and intermediate precision of the applied approaches were determined using three concentrations and three replicates of each concentration within the same day or 3 different days. Small values of the relative standard deviations gave a good indication for the high precision which characterizes these approaches (Table 4).

**Table 4:** Precision data for the determination of adapalene applying the proposed approaches

Amount taken (ng/mL)	% found	% RSD	% error
The first approach at 389 nm			
Intraday			
6.0	100.11 ± 0.85	0.85	0.49
8.0	100.55 ± 0.37	0.37	0.21
10.0	99.88 ± 0.17	0.17	0.10
Interday			
6.0	101.05 ± 1.15	1.14	0.66
8.0	99.93 ± 1.22	1.22	0.70
10.0	99.85 ± 0.55	0.55	0.32
The second approach			
SDSF at 346 nm			
Intraday			
6.0	99.67 ± 0.77	0.77	0.44
8.0	99.50 ± 0.25	0.25	0.14
10.0	100.02 ± 0.31	0.31	0.18
Interday			
6.0	100.04 ± 0.81	0.81	0.47
8.0	99.02 ± 0.97	0.98	0.57
10.0	98.28 ± 1.13	1.15	0.66
FDSF at 312.45 nm			
Intraday			
6.0	99.20 ± 0.91	0.92	0.53
8.0	100.08 ± 0.77	0.77	0.44
10.0	99.31 ± 0.42	0.42	0.24
Interday			
6.0	101.19 ± 1.37	1.35	0.78
8.0	99.74 ± 0.90	0.90	0.52
10.0	98.11 ± 1.09	1.11	0.65

#### Selectivity

The proposed approaches were found to be selective for ADP in its gel, where, satisfactory results were obtained and no interference was observed (Table 5). Moreover, the derivative synchronous fluorimetry was found to be selective for ADP in presence of its acidic and oxidative degradation products.

**Table 5:** Application of the proposed approaches for the determination of adapalene in its pharmaceutical Adapalene^®^ gel

Parameter	Amount taken (ng/mL)	First approach at 389 nm		Second approach				Comparisonmethod [3]	
		Amount found (ng/mL)	% found	Amount found (ng/mL)		% found		Amount found (µg/mL)	% found
				SDSF at 346 nm	FDSF at 312.45 nm	SDSF at 346 nm	FDSF at 312.45 nm		
Adapalene^®^ gel^a^	6.0	5.957	99.28	5.932	6.072	98.87	101.20	20.0	100.07
(0.1 % ADP)	8.0	8.082	101.03	7.922	8.026	99.03	100.33	30.0	98.80
Batch # 011371	10.0	10.007	100.07	10.022	9.905	100.22	99.05	40.0	101.03
	12.0	11.900	99.17	11.972	11.986	99.77	99.88		
Mean			99.89			99.47	100.12		99.97
S.D.			0.86			0.63	0.90		1.12
*t*			0.11			0.75	0.19		
*F*			1.69			3.11	1.56		

*N.B.* Each result is the average of three separate determinations

The tabulated *t* and *F* values are 2.57 and 9.55, respectively at *P* = 0.05 [15]

^a^Product of Borg Pharmaceutical Ind., Alexandria, Egypt

### Applications

#### Adapalene^®^ gel analysis

The present fluorimetric approaches were applied to the analysis of ADP. Four samples were determined and three replicate of each one. Satisfactory results were obtained for ADP in a good agreement with the label claims and no interference was observed (Table 5).

Statistical analysis of the results obtained by the proposed approaches and the comparison [3] method using Student’s *t* test and variance ratio F test at 95 % confidence level [15] revealed no significant difference between the performance of the approaches regarding the accuracy and precision, respectively.

#### In-vitro diffusion test

**Fig. 7:**
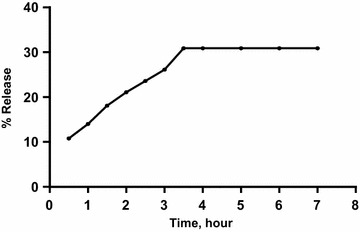
The % release of ADP from adapalene^®^ gel

The easy and excellent applicability of the first approach for the determination of ADP in its pharmaceutical gel encouraged us to conduct the in vitro diffusion test and to study the percentage of its release. In-vitro release profile of ADP from its gel in hydroethanolic solution (65 %) is shown in Fig. 7. It was found that increasing the time resulted in a subsequent increase in the % release up to 3.5 h where it reach the maximum (30 %) after which no more release was attained.

#### Stability studies

In forced degradation studies, the parent drug or the drug product is subjecting to different stress conditions. These studies play an essential role in establishing the intrinsic stability of the drug and hence help in selecting the suitable pharmaceutical dosage forms, solving the problems which may be appeared during the stages of storage and packaging. Main degradation pathways involve acidic/basic hydrolysis, oxidative, and photolytic-degradation.

According to ICH [11] guidelines, only small amount of data concerned with methodology and basics for establishing a new forced degradation study was available. Also, the required amount of the applied stress is not sufficiently discussed. Stress conditions should be realistic and not excessive. So, our target was concentrated on giving an appropriate and relevant degradation about (10–30 %) and separating the produced degradation products from the parent drug as possible.

Different forced degradation studies were tried to study the inherent stability of ADP. ADP was not susceptible to alkaline degradation as evidenced by boiling with 2 M NaOH for 2 h. On the other hand, ADP was strongly affected by acidic condition as preliminary studies showed that almost all the drug was degraded upon boiling with 1 M HCL for only 10 min. So, we directed to use 0.3 M HCl instead where 28 % of the drug was degraded after boiling for 10 min.

Similarly, ADP was susceptible to oxidative conditions and the percentage of degradation was dependent on the strength of the used hydrogen peroxide. After heating ADP with 30 % H_2_O_2_ solution at 80 °C for 10 min., about 30 % of ADP was degraded.

The impact of day light on the stability of ADP was checked after leaving its ethanolic solution on the bench in day light for 12 h and no considerable degradation was observed. Also, the solution of ADP in ethanol was exposed to UV light at two different wave lengths; 254 and 366 nm for 12 h to manifest the effect of UV light on its stability. About 25 % of ADP was degraded at the mentioned wavelengths. Unfortunately, the photolytic degradation product was not separated from the intact drug in contrast to acidic and oxidative degradation products.

#### Pathway of ADP degradation

**Scheme 1:**
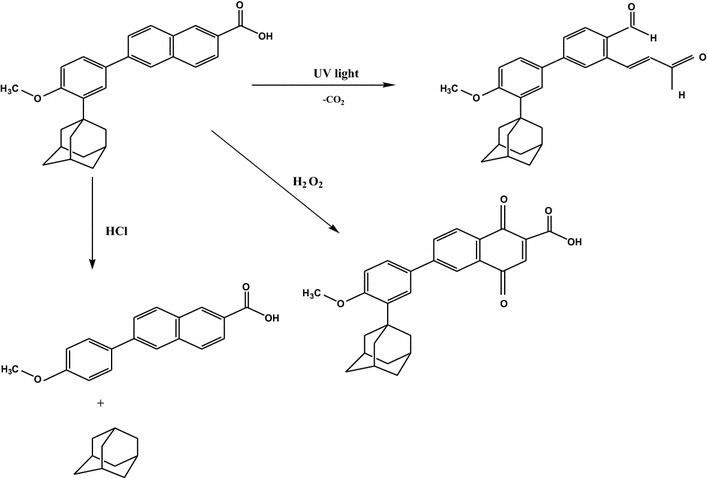
The assumed pathways of acidic, oxidative and UV light degradation of ADP

The molecular rigidity is a key element in enhancing the fluorescence behavior of many compounds as it prevents the internal conversion. Loss of rigidity of ADP is proposed under acidic stress condition via breakage of adamantine group and consequently fluorescence diminishes. As ADP being a naphthalene derivative, its photolytic irradiation may result in the degradation of naphthalene moiety into the corresponding 2-formyl cinnamaldehyde one [16, 17]. While, upon heating ADP with 30 % H_2_O_2_ it undergoes oxidative degradation with the formation of 1,4-naphthoquinone derivative. The pathway of the degradation process was proposed and postulated in Scheme 1.

## Conclusions

The proposed approaches described highly sensitive and simple fluorimetric methods for the quantitative assay of a small concentration of ADP in its pharmaceutical gel alternative to the reported sophisticated HPLC methods [3–8] or the non-sensitive derivative spectrophotometric ones [9, 10]. The conventional spectrofluorimetry was ideally suited for in vitro diffusion test. Stability studies were also conducted using different forced degradation condition according to ICH recommendation. Fortunately, second and first derivative synchronous fluorimetry were capable to resolve the band of ADP from its acidic and oxidative degradation products at 346 and 312.45 nm after recording the synchronous fluorimetry using Δ λ of 80 and 100 nm, respectively. Consequently, the stability indicating power of this approach can be assessed.

## Abbreviations

ADP: adapalene
LOD: limit of detection
LOQ: limit of quantitation
ICH: International Conference on Harmonization
